# Agonistic autoantibodies against ß2-adrenergic receptor influence retinal microcirculation in glaucoma suspects and patients

**DOI:** 10.1371/journal.pone.0249202

**Published:** 2021-05-07

**Authors:** Bettina Hohberger, Sami Hosari, Gerd Wallukat, Rudolf Kunze, Johann Krebs, Meike Müller, Till Hennig, Robert Lämmer, Folkert Horn, Luis E. Muñoz, Martin Herrmann, Christian Mardin

**Affiliations:** 1 Department of Ophthalmology, University of Erlangen-Nürnberg, Friedrich-Alexander-University of Erlangen-Nürnberg, Erlangen, Germany; 2 Max Delbrück Center for Molecular Medicine, Berlin, Germany; 3 Science Office, Berlin-Buch, Campus Max Delbrück Center for Molecular Medicine, Berlin, Germany; 4 Department of Internal Medicine 3—Rheumatology and Immunology, University of Erlangen-Nürnberg, Friedrich-Alexander University (FAU) Erlangen-Nürnberg, Germany; Bascom Palmer Eye Institute, UNITED STATES

## Abstract

**Purpose:**

Agonistic β2-adrenergic receptor autoantibodies (β2-agAAb) have been observed in sera of patients with ocular hypertension and open-angle glaucoma (OAG). They target the β2-receptors on trabecular meshwork, ciliary body and pericytes (Junemann et al. 2018; Hohberger et al. 2019). In addition to their influence on the intraocular pressure, an association to retinal microcirculation is discussed. This study aimed to investigate foveal avascular zone (FAZ) characteristics by en face OCT angiography (OCT-A) in glaucoma suspects and its relationship to β2-agAAb status in patients with OAG.

**Material and methods:**

Thirty-four patients (28 OAG, 6 glaucoma suspects) underwent standardized, clinical examination including sensory testing as white-on-white perimetry (Octopus G1, mean defect, MD) and structural measures as retinal nerve fibre layer (RNFL) thickness, neuroretinal rim width (BMO-MRW), retinal ganglion cell layer (RGCL) thickness, and inner nuclear layer (INL) thickness with high-resolution OCT. FAZ characteristics were measured by OCT-A scans of superficial vascular plexus (SVP), intermediate capillary plexus (ICP), and deep capillary plexus (DCP). FAZ-R was calculated (area FAZ (SVP)/area FAZ (ICP)). Using cardiomyocyte bioassays we analysed serum samples for the presence of β2-agAAb.

**Results:**

(I) Total mean FAZ area [mm^2^]: 0.34±0.16 (SVP), 0.24±0.12 (ICP), and 0.49±0.24 (DCP); mean FAZ-R 1.58±0.94. No correlation was seen for FAZ-R with MD, RNFL, BMO-MRW, RGCL thickness and INL thickness (p>0.05). (II) ß2-agAAb have been observed in 91% patients and showed no correlation with MD, RNFL, BMO-MRW, RGCL thickness and INL thickness (p>0.05). (III) FAZ-R correlated significantly with the β2-agAAb-induced increase of the beat rate of cardiomyocyte (p = 0.028).

**Conclusion:**

FAZ characteristics did not correlate with any glaucoma associated functional and morphometric follow-up parameter in the present cohort. However, level of β2-agAAb showed a significantly correlation with FAZ-ratio. We conclude that β2-agAAb might be a novel biomarker in glaucoma pathogenesis showing association to FAZ-ratio with OCT-A.

## Introduction

Glaucoma is known as a multifactorial neurodegenerative disease. The exact pathomechanism is still elusive. Thus, several factors seem to act and interact within this neurodegenerative disorder. Next to an increased intraocular pressure (IOP), being the main risk factor, e.g. oxidative stress [1], trace elements [2], an altered trabecular meshwork [3], immunological [4] and neuroinflammatory processes [5] are involved. In addition vascular dysregulation was seen as pathogenetic agent [6]. It is supposed that due to an insufficient nutritive support of retinal ganglion cells via altered microcirculation neurodegeneration could be promoted. A restricted autoregulation with additional vasospasms may enhance this pathophysiologic changes [7].

**Fig 1:**
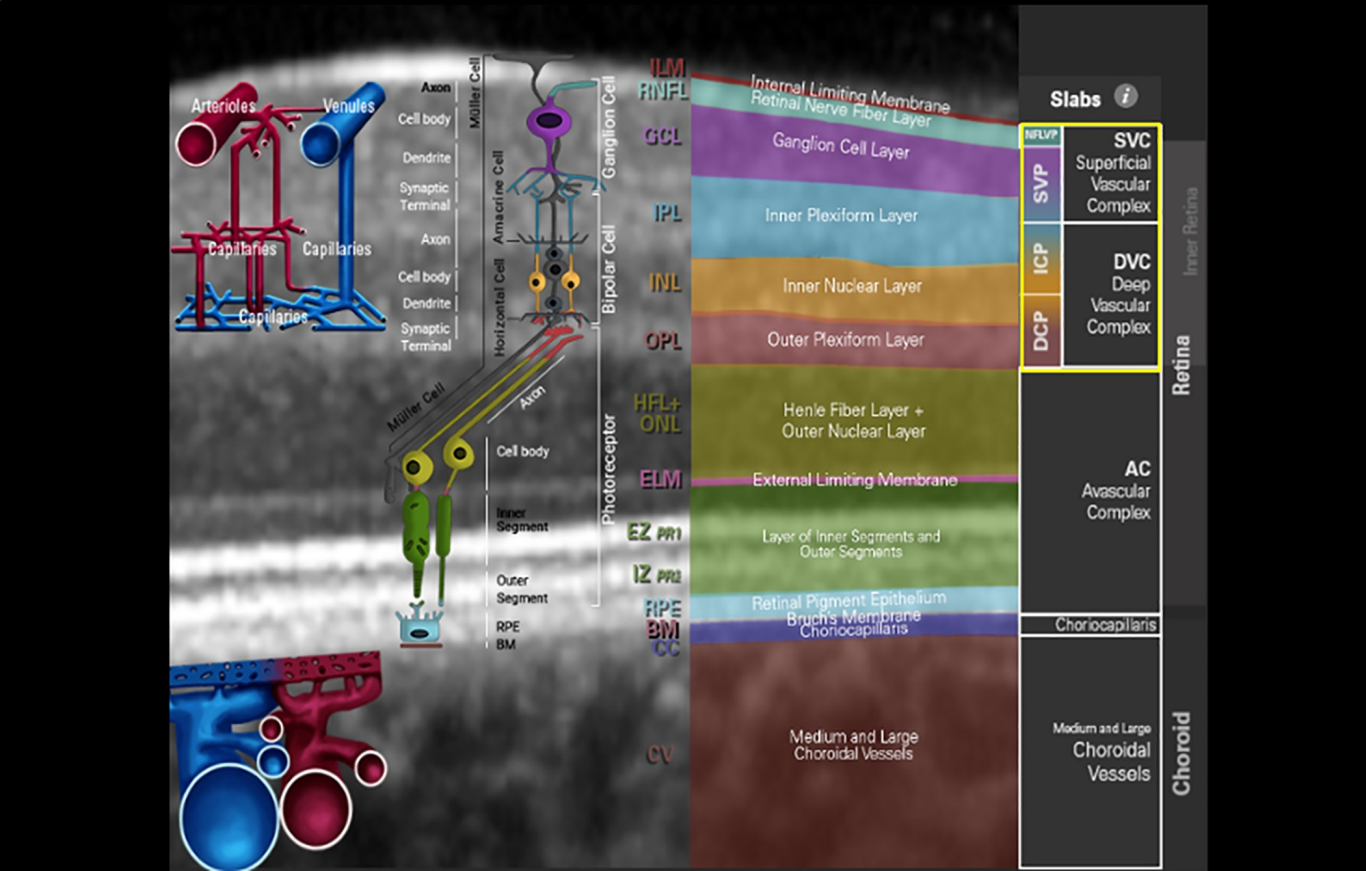
Structure OCT detail of the macula with the corresponding structural OCT layers and OCT-A slabs of the inner retina (yellow box). Image courtesy by Heidelberg Engineering, Heidelberg, Germany.

OCT-angiography (OCT-A) offers the diagnostic option to visualize and quantify retinal microcirculation with subsequent software tools (e.g. Erlangen Angio Tool [8]). Heidelberg Spectralis II OCT-A (Heidelberg, Germany) is able to scan macular microcirculation in three layers: superficial vascular plexus (SVP, thickness: 80 μm), intermediate capillary plexus (ICP, thickness: 50μm), and deep capillary plexus (DCP, thickness: 40 μm). These layers correspond well with anatomical structures [9]: SVP corresponds mainly to the ganglion cell layer (GCL), ICP to the inner plexiform layer (IPL) and inner nuclear layer (INL), and DCP mainly to the outer plexiform layer (OPL, Fig 1).

Glaucoma damage focuses on GCL loss, thus, we hypothesize that changes in retinal microcirculation might be accented in SVP and adjacent ICP, potentially most obvious in foveal avascular zone characteristics (FAZ) [10]. A potential link between microvascular retinal alterations and immunological findings in glaucoma might be mirrored by specific agonistic autoantibodies against the ß2-adrenoceptors (ß2-agAAb), as ß2-adrenoreceptors (ß2-AR) are present on pericytes of retinal microvessels [11]. We observed these ß2-agAAb in a high percentage in sera of patients with primary (POAG), secondary open-angle glaucoma (SOAG) or ocular hypertension (OHT), yet not in controls [12, 13]. The target of ß2-agAAb is additionally present on cells of the trabecular meshwork (TM) and ciliary body [14, 15], both involved in regulation of IOP. Thus, it is assumed that ß2-agAAb might be involved in the pathological increase of IOP via rise of outflow resistance in the TM and increase of production of aqueous humor in the ciliary body. The hypothesis of an increase of outflow resistance is supported by data of Putney et al., describing an increased volume of trabecular meshwork cells in patients with glaucoma. The altered cell volume was reportedly induced by osmotic changes due to Na^+^-K^+^-Cl^—^cotransporters, activated by ß-adrenergic stimulation [16]. Clinical data of a proof-of-principle study supported the hypothesis of a potential involvement of ß2-agAAb in glaucoma pathogenesis, as IOP showed a transient decrease after immunadsorption of all immunoglobulins including ß2-agAAb [12].

The aim of the present study was to investigate foveal avascular zone (FAZ) characteristics of SVP, ICP, and DCP by en face OCT-A in glaucoma suspects and open-angle glaucoma patients (OAG) in relation to their corresponding β2-agAAb status.

## Materials and methods

### Patients

Thirty-four patients were recruited from the Department of Ophthalmology and Eye Hospital, Friedrich-Alexander-University Erlangen-Nürnberg (Erlangen Glaucoma Registry, ISSN 2191-5008, CS-2011; NTC00494923 [17]): 6 glaucoma suspects (4 ocular hypertension, OHT; 2 pre-perimetric OAG), 28 patients with OAG. Demographic data for group and subgroups are displayed in Table 1.

**Table 1:** Demographic data for total cohort and subdivided into glaucoma suspects and glaucoma patients: Age, gender, intraocular pressure at the day of examination with glaucoma therapy (IOP), number of antiglaucomatous eye drops, best corrected visual acuity (BCVA): Mean±SD; number family history of glaucoma, diabetes, hypertension.

	Total	Glaucoma suspects	Patients with OAG
**Age**	67.1±12.0	69.5±14.0	66.6±12.0
**Gender (female/male)**	16/18	2/4	14/14
**IOP**	15.0±3.9	13.5±2.4	15.3±4.1
**Number of antiglaucomatous eye drops**	1.4±1	1.7±1	1.4±1
**BCVA**	0.8±0.18	0.85±0.08	0.84±0.20
**Family history for glaucoma**	17	1	16
**Diabetes**	1	0	1
**Hypertension**	20	3	17

All patients underwent a standardized ophthalmological examination, which included slit-lamp biomicroscopy, funduscopy and Goldmann applanation tonometry. Additionally, white-on-white perimetry (Octopus 500, program G1, Interzeag, Schlieren, Switzerland; mean defect (MD), n = 34) and measurements of the global retinal nerve fiber layer (RNFL, n = 33), Bruchs’ membrane opening—minimum rim width (BMO-MRW, n = 33), retinal ganglion cell layer (RGCL, n = 33) and inner nuclear layer (INL, n = 33) were done (Heidelberg OCT II Spectralis, version 1.9.10.0, Heidelberg Engineering, Heidelberg, Germany). Diagnosis of glaucoma suspects and POAG were based according to the criteria of the Erlangen Glaucoma registry [17]. Glaucomatous optic disc was classified after Jonas [18]. If both eyes met the inclusion criteria, data of one eye were chosen randomly for statistical analysis. Every participant provided informed written consent. The study protocol was performed in accordance with the tenets of the Declaration of Helsinki and was approved by the local ethics committee of the university of Erlangen-Nürnberg.

### Glaucoma suspects

Diagnosis of glaucoma suspects was based on a repeated (≥2 times) increased IOP > 21 mmHg. Visual fields showed no glaucomatous scotoma. The optic disc was with (diagnosis: pre-perimetric OAG, classified as proposed by Jonas, [18]) or without glaucomatous alterations (diagnosis: OHT).

### POAG

The diagnosis of POAG was based on an open anterior chamber angle. IOP had to be increased (> 21 mmHg), repeated ≥2 times. The optic disc showed glaucomatous alterations according to the classification after Jonas [18]. Visual field yielded glaucomatous scotoma (confirmed at least once): ≥3 adjacent test points having a deviation ≥ 5 dB and with one test point with a deviation > 10 dB lower than normal or ≥2 adjacent test points with a deviation ≥ 10 dB or at least 3 adjacent test points with a deviation ≥ 5 dB abutting the nasal horizontal meridian or a mean visual field defect of > 2.6 dB.

### Cardiomyocyte bioassay

For the functional experiments we used neonatal rat cardiomyocytes in primary culture. The cells were prepared freshly every week with the permission 49004/19 of the Max Delbrück Centre for Molecular Medicine. Cell culture of cardiac myocytes derived from heart ventricle of 1–2 day-old Sprague-Dawley rats [19]. We included this information into the revised version of the manuscript. After digestion with a 0.25% solution of crude porcine trypsin (Serva, Germany), the cells were dispersed and suspended in a SM20-I medium (Biochrom, Germany), glutamine (Serva, Germany), containing penicillin (Heyl, Germany), streptomycin (HEFA Pharma; Germany), 10% heat-inactivated neonatal calf serum (Gibco, Germany), hydrocortisone (Merck, Germany), and fluorodeoxyuridine (Serva, Germany). The cardiomyocyte cells were seeded with a field density of 160.000 cells/cm^2^. The culture medium was refaced after 24 hours. Previous to stimulation the cells were cultured for 3–4 days at 37°C. The medium was renewed with fresh culture solution 2 hours before start of the experiments. Beating rate was counted of 7–10 selected spontaneously beating cardiomyocte or synchronously contracting cell clusters per flask for 15 seconds, which were placed on a heated stage of an inverted microscope at 37°C (cut-off < 2.0 Units, U). The cardiomyocytes were incubated with the immunoglobulin fractions of each probands’ sera (duration: 60 min; dilution of 1:40). The change in beating rate after incubation with each probands’s sera was measured, respectively. 1 Unit (U) reflects 4 beats/min. Cutoff was 2 U.

### OCT-angiography

We performed en face OCT-A measurements employing a Heidelberg OCT II Spectralis device (Heidelberg, Germany). The OCT-A scan recorded a retinal slab of 2.9 mm x 2.9 mm with a 15° angle and a lateral resolution of 5.7 μm/pixel. Data were exported and analyzed by the Erlangen Angio-Tool (EA-Tool, Matlab; The MathWorks, Inc., R2017b) [20]. EA-Tool used binariziation by using the Otsu thresholding algorithm [21] and Frangi vesselness filter [22]. En face OCT-A application in combination with EA-Tool allowed analysis of FAZ area in three different layers: superficial vascular plexus (SVP), intermediate capillary plexus (ICP), and deep capillary plexus (DCP). We present FAZ data for SVP, ICP and DCP, respectively.

### Statistical analysis

Statistical analysis was done using SPSS (version 21.0) and Excel (version 2010). The FAZ-to-FAZ-ratio (FAZ-R) between FAZ of SVP and ICP was calculated. Data were presented by mean ± standard deviation and percentage. T-test and correlation analysis were performed.

## Results

### OCT-angiography

**Fig 2:**
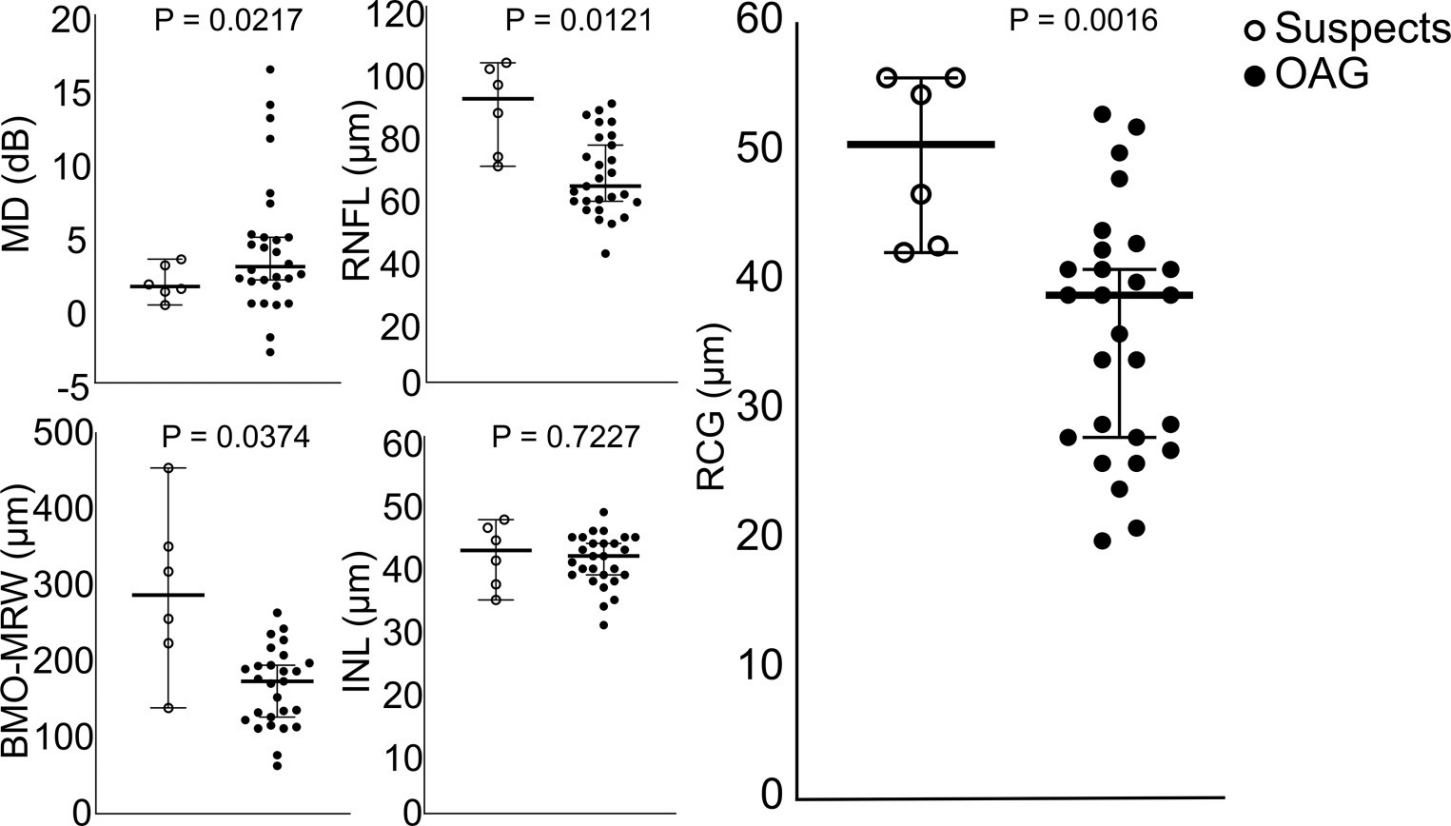
Perimetric and morphometric data of total, glaucoma suspects and patients with open-angle glaucoma (OAG): Mean defect (MD), retinal nerve fiber (RNF), Bruchs’ membrane opening—minimum rim width (BMO-MRW), retinal ganglion cell (RGC), inner nuclear layer (INL).

Functional and morphometric data of Spectralis OCT II measurements are displayed in Fig 2.

OCT-A data of all patients showed a mean FAZ of 0.34±0.16 mm^2^ (range: 0.13 mm2–0.92 mm^2^) in SVP, 0.24±0.12 mm^2^ (range: 0.08 mm2–0.59 mm^2^) in ICP, and 0.49±0.24 mm^2^ (range: 0.18 mm^2^–1.09 mm^2^) in DCP. FAZ-R yielded a mean of 1.58±0.94.

Subgroup analysis showed a mean FAZ of 0.21±0.04 mm^2^ (range: 0.13 mm2–0.26 mm^2^) in SVP, 0.12±0.05 mm^2^ (range: 0.08 mm2–0.21 mm^2^) in ICP, and 0.29±0.06 mm^2^ (range: 0.22 mm^2^–0.37 mm^2^) in DCP in glaucoma suspects. FAZ-R of glaucoma suspects yielded a mean of 1.81±0.63. Patients with OAG showed a mean FAZ of 0.36±0.17 mm^2^ (range: 0.17 mm2–0.92 mm^2^) in SVP, 0.26±0.12 mm^2^ (range: 0.12 mm2–0.59 mm^2^) in ICP, and 0.53±0.24 mm^2^ (range: 0.18 mm^2^–1.09 mm^2^) in DCP. Additionally, FAZ-R yielded a mean of 1.53±0.99.

No significant correlation was seen for FAZ-R with MD (p>0.05), RNF thickness (p>0.05), BMO-MRW (p>0.05), RGC thickness (p>0.05), and INL thickness (p>0.05) for group (total cohort) and subgroup analysis (glaucoma suspects, OAG), respectively.

### β2-agAAb seropositivity

**Fig 3:**
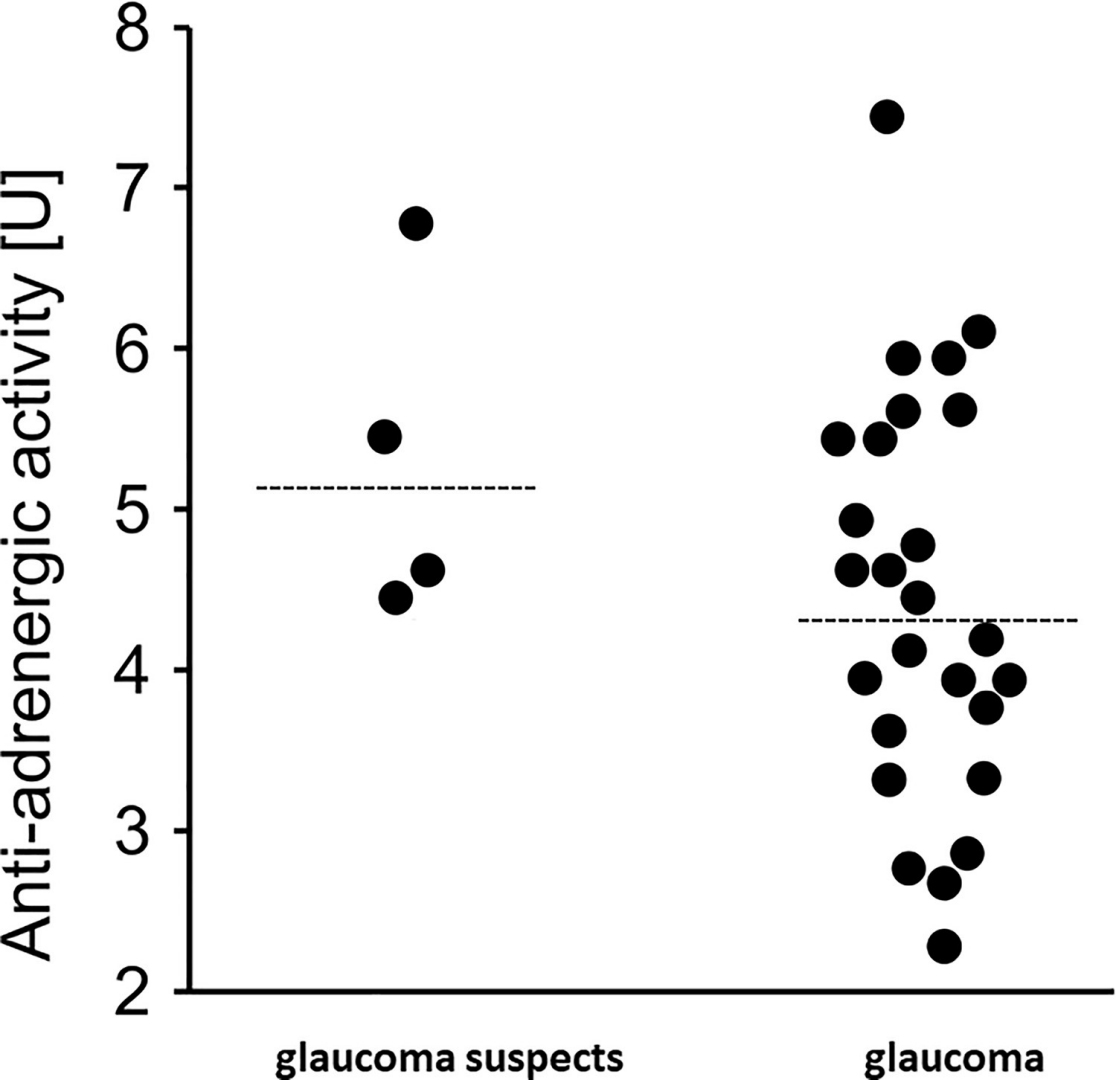
Anti-adrenergic activity β2-agAAb [U] subdivided in glaucoma suspects and glaucoma patients (raw data; median).

A β2-agAAb seropositivity was observed in 91% of all patients with a mean increase of beat in comparison to the basal beating rate of 4.5±1 U (range: 2.3–7.5 U, Fig 3). Only 9% of all patients turned out to be seronegative for β2-agAAb (<2.0 U).

Primary functional and morphometric data considering β2-agAAb seropositivity are presented in Table 2.

**Table 2:** Perimetric and morphometric data considering β2-agAAb seropositivity: Mean defect (MD), retinal nerve fiber (RNFL), Bruchs’ membrane opening—minimum rim width (BMO-MRW), retinal ganglion cell (RGCL), inner nuclear layer (INL).

	β2-agAAb seropositive patients	β2-agAAb seronegative patients
**MD** [dB]	3.7±4.0	4.9±7.8
**RNFL** [μm]	71.3±14.9	57.0±15.4
**BMO-MRW** [μm]	194.3±80.7	128.0±47.5
**RGCL** [μm]	39.6±9.9	27.7±10.0
**INL** [μm]	40.4±4.0	41.0±7.6

**Fig 4:**
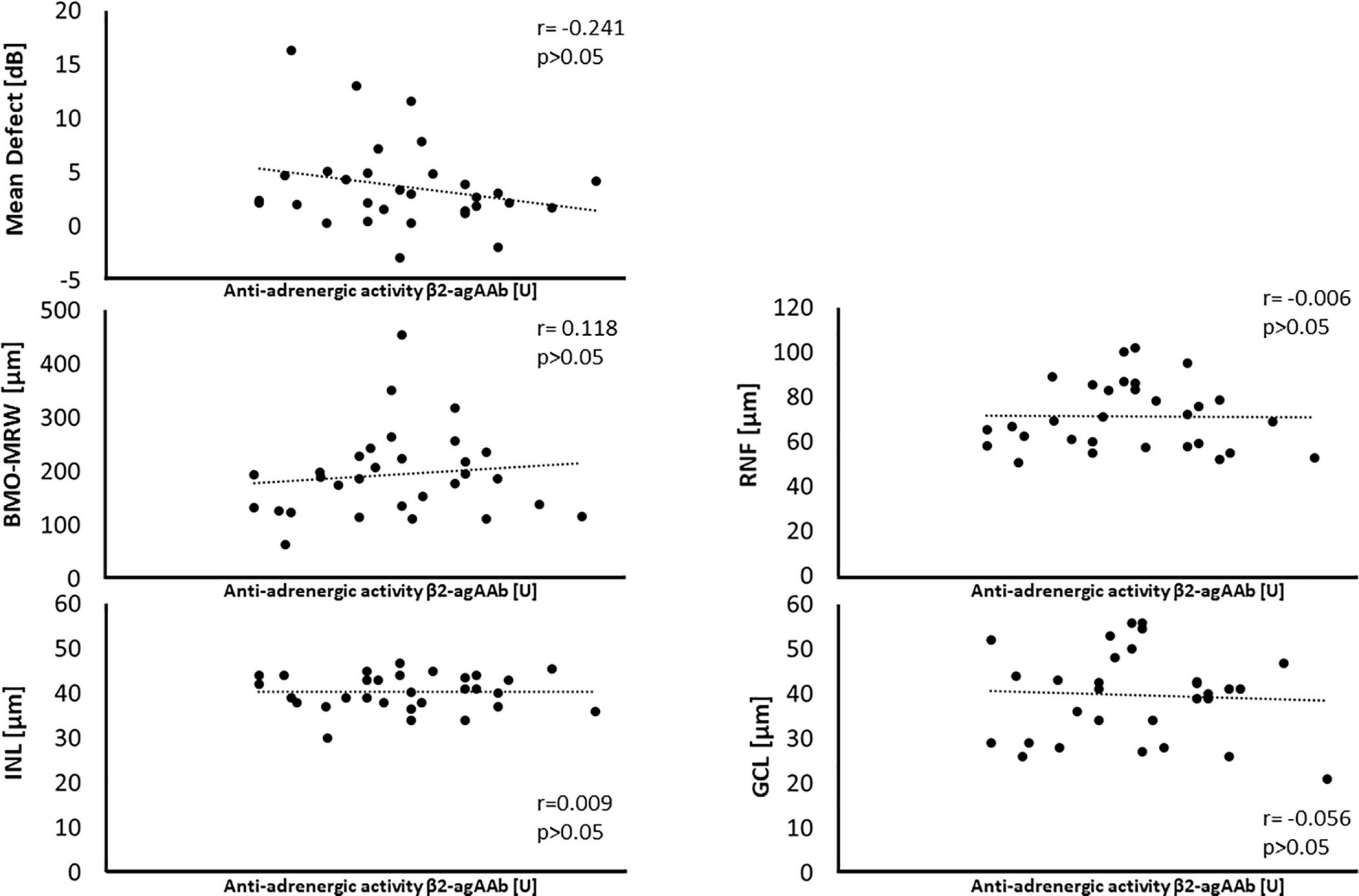
Correlation plots of anti-adrenergic activity β2-agAAb [U] and glaucoma follow-up parameters (mean defect, MD; Bruchs’ membrane opening—minimum rim width, BMO-MRW; inner nuclear layer, INL; retinal nerve fiber, RNF; retinal ganglion cell, RGC): We observed no significant correlations for all presented parameters (p>0.05, Pearson’s correlation).

β2-agAAb seropositive patients showed no correlation of β2-agAAb beat rate with MD (p>0.05), RNF thickness (p>0.05), BMO-MRW (p>0.05), RGC thickness (p>0.05) and INL thickness (p>0.05) for group (total cohort) and subgroup (glaucoma suspects, OAG) analysis, respectively (Fig 4).

Considering β2-agAAb seropositivity a mean FAZ of 0.34±0.17 mm^2^ (range: 0.13 mm2–0.92 mm^2^) was observed in β2-agAAb seropositive and 0.32±0.20 mm^2^ (range: 0.20 mm2–0.55 mm^2^) in β2-agAAb seronegative patients for the SVP layer. Data of ICP showed a mean FAZ of 0.24±0.13 mm^2^ (range: 0.08 mm^2^–0.59 mm^2^) in β2-agAAb seropositive and 0.27±0.09 mm^2^ (range: 0.21 mm^2^–0.37 mm^2^) in β2-agAAb negative patients. FAZ of DCP was 0.48±0.22 mm^2^ (range: 0.18 mm2–1.09 mm^2^) in β2-agAAb seropositive and 0.56±0.42 mm^2^ (range: 0.25 mm2–1.03 mm^2^) in β2-agAAb negative patients. We observed a mean FAZ-R of 1.62±0.97 in β2-agAAb seropositive and 1.13±0.31 in β2-agAAb negative patients.

**Fig 5:**
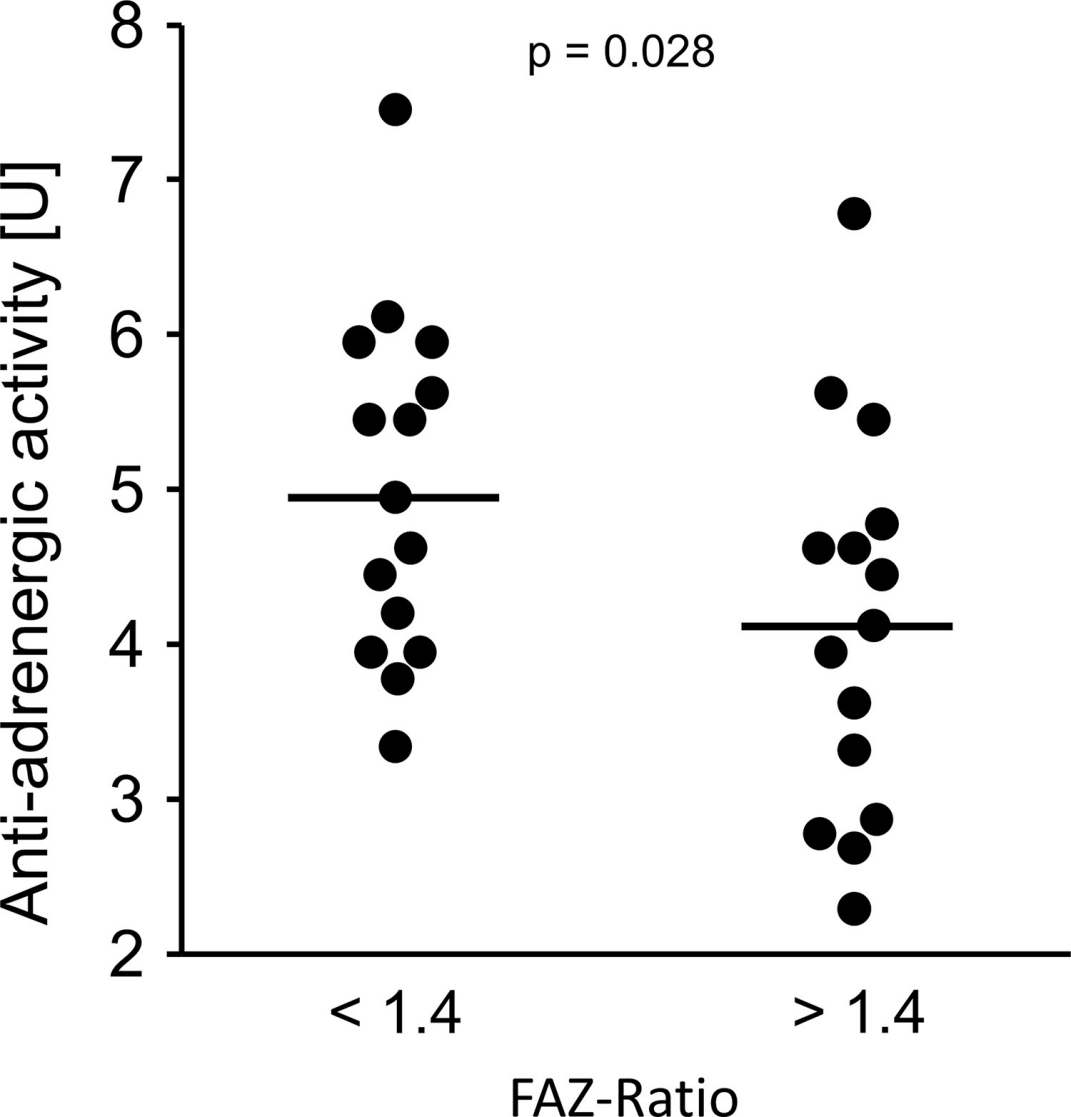
Anti-adrenergic activity β2-agAAb [U] considering FAZ-ratio (cutoff 1.4; p = 0.028), plotted as raw data and median.

A significant correlation of β2-agAAb beat rate with FAZ-R was observed (p = 0.028, Fig 5).

## Discussion

Agonistic autoantibodies can be found in several diseases with a common vascular component [19, 23–26]. β2-agAAb, able to activate the adrenergic ß2-AR have recently been reported in patients with POAG, SOAG and even OHT, yet not in persons with healthy eyes [12, 13] Thus, we assume that β2-agAAb is involved in the etiology glaucoma by a common mechanism. A previous study offered an involvement of these β2-agAAb in the regulation of IOP [12]. ß2-AR expressed on pericytes, on the cells of the ciliary body and the trabecular meshwork [14, 15] represent potential molecular targets that cause the clinical findings. Thus, the present study aimed to investigate a potential involvement of β2-agAAb on retinal microcirculation in patients with glaucoma. Ninety-one percent of all patients were seropositive for ß2-agAAb, not correlating with standard glaucoma follow-up parameters (MD, RNFL, BMO-MRW, RGCL-, INL thickness). Analysis of FAZ in the three different macula layers yielded that FAZ-R correlated significantly with β2-agAAb beat rate in β2-agAAb seropositive patients, yet not with MD, RNF thickness, BMO-MRW, RGC thickness, and INL thickness.

OCT angiography is a novel diagnostic tool to visualize the retinal microcirculation of the macula and the peripapillary region. Invasive techniques (e.g. fluorescence angiography) required the application of dyes with potential side effects. As non-invasive method OCT-A does not include dyes and thus offers a basis for subsequent research issues. A motion contrast algorithm with high spatial resolution yields an improvement of the three-dimensional image of the retinochoroidal vasculature. In the beginning retinal microvasculature could be resolved into 2 different layers (superficial vascular plexus (SVP) and deep capillary plexus (DCP)) [27] with current improvements up to 3 layers in the latest OCT-A tools (SVP, ICP, DCP) [28]. Experimental studies showed a good correlation of these layers with anatomical findings [9]. Spectralis II OCT-A uses a probabilistic full-spectrum amplitude decorrelation algorithm (FSADA), reducing the signal-to-noise ratio with improvement of axial resolution (3.9 μm/pixel) [29]. Additional implementation of projection artefact removal (PAR) tool allows analysis of deeper retinal microvasculature without overlying artefacts. As no commercial software was available for analysis of OCT-A images, a custom made Erlangen-Angio-Tool (EA-Tool) was coded. EA-tool uses a Frangi’s vesselness filter for convolving [30] and an Otsu’s thresholding algorithm for binarization of the image raw data [21]. As previous data showed an even good reliability for the EA-Tool [20] this was the basis for its use in clinical and research settings.

Clinical studies identified an altered retinal microcirculation in a wide range of retinochoroidal diseases (e.g. diabetic retinopathy, vasculitis, age-related maculopathy) [31–35]. Even glaucoma patients’ microcirculation was seen to be impaired in OCT-A imaging (see review [36, 37]). Significantly different vessel densities were observed in glaucoma patients when compared to healthy controls with good areas under the receiver operating characteristic curve (AUC) [38, 39]. Interestingly, in early glaucoma diagnosis OCT-A data were hypothesized to be better diagnostic tools than retinal nerve fiber layer thickness [40]. The present study showed that especially the differences between the three retinochoroidal layers might be of interest in early glaucoma diagnosis. The ratio of SVP to ICP confirmed this as this FAZ-R did not correlate with canonical glaucoma follow-up parameters (MD, RNF thickness, BMO-MRW, RGC thickness, and INL thickness). However, it correlated with the levels of β2-agAAb. We detected these autoantibodies in patients with early glaucoma disease or OAG [12]. Thus, we propose that parameters (β2-agAAb, FAZ-R), which are present in very early stages of glaucoma pathogenesis might offer a novel, early option for an extended glaucoma diagnosis. As they correlate by each other, yet not with standard glaucoma parameters, it might be that β2-agAAb and FAZ-R recruit a further molecular pathway, which does not influence the canonical glaucoma follow-up parameters. As we observed a correlation of FAZ ratio with the β2-agAAb beat rate in the present study and a seropositivity of β2-agAAb even in eyes with normal RFNL and normal RGCL (i.e. ocular hypertension) in a previous study [12], we hypothesize that β2-agAAb are present very early in glaucoma pathogenesis and are linked to microcirculation.

**Fig 6:**
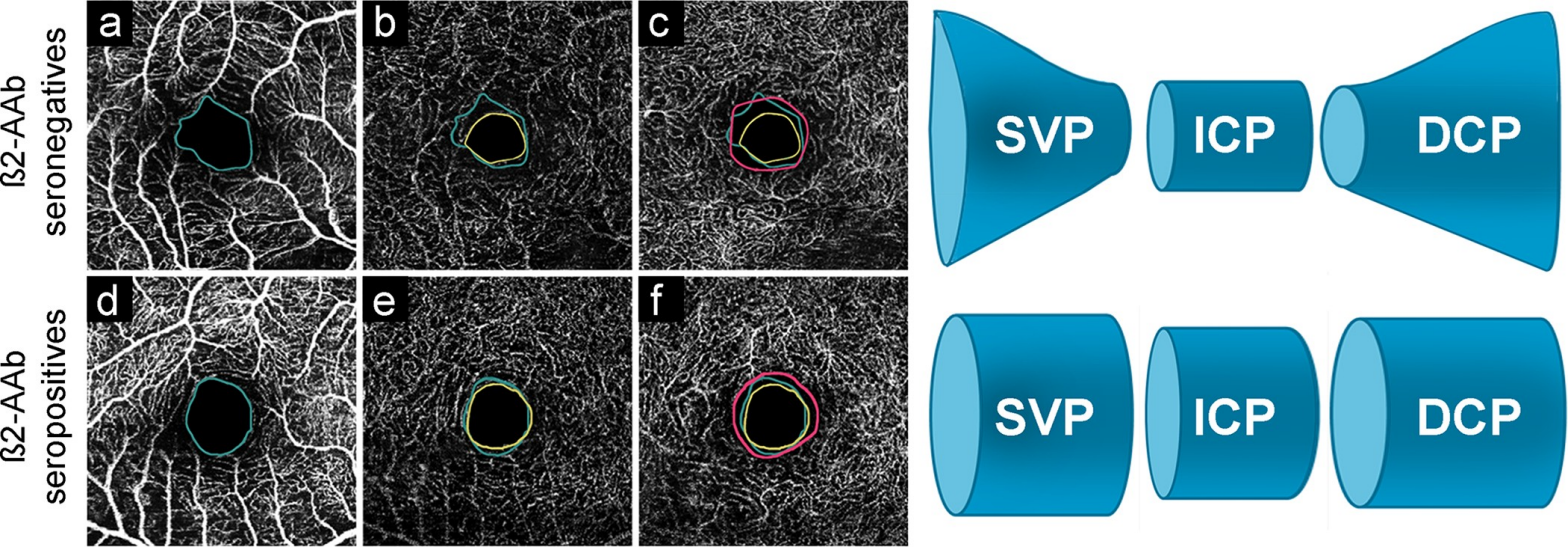
FAZ of superficial vascular plexus (SVP; green; a, d), intermediate capillary plexus (ICP; yellow; b, e), and deep capillary plexus (DCP; pink; c, f) in a β2-agAAb seronegative (a-c) and seropositive (d-f) OAG patient including a schematic sketch of the FAZ of SVP, ICP, DCP considering β2-agAAb seropositivity.

β2-agAAb are frequent. Patients with POAG and SOAG showed β2-agAAb in >78% of the patients [13, 41] β2-agAAb “hyperactivate” the adrenergic β2-AR in various tissues (e.g. ciliary body, and trabecular meshwork) with consecutive influence on IOP via hypersecretion of aqueous humor and decrease of the outflow facility [12]. Activation of β2-and β3-AR on retinal blood vessels reportedly induce vasodilatation [42, 43]. A ‘direct’ autonomic innervation is not present in retinal blood vessels [44]. Yet, sympathetic activation can activate β2-AR ‘indirectly’ via its transmitter adrenaline [42]. Additionally, β2- and β3-AR agonists reportedly mediate retinal vasodilatation [45]. Considering the present data available in literature, β2-AR mediated vasodilation is an important factor, regulating the retinal microcirculation. To the best of our knowledge, the exact molecular pathway mediated by β2-AR in retinal vessels is still elusive. It was reported that in ischemia the behavior of β2-AR varied: whereas a subgroup was internalized, a second group showed an alternate desensitization that finally caused an increase in affinity [46]. This in vitro observation supports our hypothesis of a link between β2-agAAb (via β2-AR) and impaired retinal microcirculation in glaucoma eyes. As FAZ-R significantly correlated with β2-agAAb beat rate (p = 0.028), the present data show that the size of different retinal microvascular layers was mutually altered in the presence of β2-agAAb and resulted in similar FAZ sizes of both microvascular layers (Fig 6).

If the ‘lacking’ vessels are really missing or even not perfused remains unknown. The present study suggests that β2-agAAb influence the retinal microcirculation. We hypothesize that the influence β2-agAAb on FAZ-R is mediated by the prolonged activation of the β2-receptor. Contrary to canonical agonists, activating the β2-receptor only for a short-term, agAAb are known to mediate a stable and even prolonged signal. This uncontrolled hyperactivation of the G-protein coupled receptor can result in cell-toxic effects, as observed for agAAb against ß1AR (cardiotoxic effect) in a rat model [24]. Differences or ratios between FAZ of the distinct microvascular layers detected in OCT-A are sensible markers for early pathogenetic alterations. Retinal microcirculation should be considered a single nutritional unit. Thus, pathological alterations in one of the layers of the microvascular most likely influence the other ones.

The present study is not without limitations. Study size is small (n = 34), yet data should be seen as having a pilot character and should be confirmed in subsequent studies. Considering the number of subjects per group (β2-agAAb positive vs. negative), we did not make a group comparison. Yet, statistically significant correlations between FAZ-R and β2-agAAb were already observed in this small patient group. Furthermore, the study focused on changes of FAZ in glaucoma suspects and patients with OAG taking into account the status of β2-agAAb. Subsequent research will investigate potential alterations in vessel density of SVP, ICP and DCP in the presence of β2-agAAb.

## Conclusion

FAZ characteristics of the macula showed a significantly correlation with β2-agAAb beat rate in glaucoma suspects and patients with open-angle glaucoma. These data support the hypothesis of a pathological role of β2-agAAb in the microcirculation of the macula. Level of β2-agAAb did not correlate with the canonical glaucoma follow-up parameters. Yet, with FAZ characteristics, β2-agAAb should be considered a novel risk factor of glaucoma. In addition, FAZ-R should be seen as a novel early diagnostic morphological marker of glaucoma disease.
